# Influence of Macrocyclic Chelators on the Targeting Properties of ^68^Ga-Labeled Synthetic Affibody Molecules: Comparison with ^111^In-Labeled Counterparts

**DOI:** 10.1371/journal.pone.0070028

**Published:** 2013-08-01

**Authors:** Joanna Strand, Hadis Honarvar, Anna Perols, Anna Orlova, Ram Kumar Selvaraju, Amelie Eriksson Karlström, Vladimir Tolmachev

**Affiliations:** 1 Unit of Biomedical Radiation Sciences, Rudbeck Laboratory, Uppsala University, Uppsala, Sweden; 2 Division of Protein Technology, School of Biotechnology, KTH Royal Institute of Technology, Stockholm, Sweden; 3 Preclinical PET Platform, Department of Medicinal Chemistry, Uppsala University, Uppsala, Sweden; Genentech, United States of America

## Abstract

Affibody molecules are a class of small (7 kDa) non-immunoglobulin scaffold-based affinity proteins, which have demonstrated substantial potential as probes for radionuclide molecular imaging. The use of positron emission tomography (PET) would further increase the resolution and quantification accuracy of Affibody-based imaging. The rapid in vivo kinetics of Affibody molecules permit the use of the generator-produced radionuclide ^68^Ga (T_1/2_ = 67.6 min). Earlier studies have demonstrated that the chemical nature of chelators has a substantial influence on the biodistribution properties of Affibody molecules. To determine an optimal labeling approach, the macrocyclic chelators 1,4,7,10-tetraazacylododecane-1,4,7,10-tetraacetic acid (DOTA), 1,4,7-triazacyclononane-N,N,N-triacetic acid (NOTA) and 1-(1,3-carboxypropyl)-1,4,7- triazacyclononane-4,7-diacetic acid (NODAGA) were conjugated to the N-terminus of the synthetic Affibody molecule Z_HER2:S1_ targeting HER2. Affibody molecules were labeled with ^68^Ga, and their binding specificity and cellular processing were evaluated. The biodistribution of ^68^Ga-DOTA-Z_HER2:S1,_^68^Ga-NOTA-Z_HER2:S1_ and ^68^Ga-NODAGA-Z_HER2:S1_, as well as that of their ^111^In-labeled counterparts, was evaluated in BALB/C nu/nu mice bearing HER2-expressing SKOV3 xenografts. The tumor uptake for ^68^Ga-DOTA-Z_HER2:S1_ (17.9±0.7%IA/g) was significantly higher than for both ^68^Ga-NODAGA-Z_HER2:S1_ *(*16.13±0.67%IA/g) and ^68^Ga-NOTA-Z_HER2:S1_ (13±3%IA/g) at 2 h after injection. ^68^Ga-NODAGA-Z_HER2:S1_ had the highest tumor-to-blood ratio (60±10) in comparison with both ^68^Ga-DOTA-Z_HER2:S1_ (28±4) and ^68^Ga-NOTA-Z_HER2:S1_ (42±11). The tumor-to-liver ratio was also higher for ^68^Ga-NODAGA-Z_HER2:S1_ (7±2) than the DOTA and NOTA conjugates (5.5±0.6 vs.3.3±0.6). The influence of chelator on the biodistribution and targeting properties was less pronounced for ^68^Ga than for ^111^In. The results of this study demonstrate that macrocyclic chelators conjugated to the N-terminus have a substantial influence on the biodistribution of HER2-targeting Affibody molecules labeled with ^68^Ga.This can be utilized to enhance the imaging contrast of PET imaging using Affibody molecules and improve the sensitivity of molecular imaging. The study demonstrated an appreciable difference of chelator influence for ^68^Ga and ^111^In.

## Introduction

Malignant transformation of cells is often associated with an unusual expression of certain types of cell-surface proteins, e.g. receptors, cell adhesion molecules or proteins active in embryonic development. Molecular recognition of these proteins by e.g. monoclonal antibodies can be used in targeted therapy for specific treatment of malignant cells. The major problem is that a particular molecular target is expressed only in a fraction of the tumors of a certain origin, and only patients with such tumors would benefit from targeted therapy. Moreover, a molecular target expression might be dissimilar in primary tumor and in different metastases in the same patient [1]. Furthermore, expression of the target might be changed during the natural course of disease or in response to therapy [2]. In vivo molecular imaging can be a helpful clinical tool for identification of patients eligible for a particular targeted therapy [3]–[4]. A new class of affinity ligands, Affibody molecules, has previously demonstrated very promising properties as probes for radionuclide molecular imaging [5]. Affibody molecules are affinity proteins derived from the immunoglobulin-binding B domain of staphylococcal protein A. The small size (6–7 kDa) and high affinity (dissociation constants, K_D_, in low nanomolar or sub-nanomolar range) make them good candidates for molecular imaging probes [6]. The use of Affibody molecules instead of antibodies provides such advantages as more rapid extravasation, efficient tumor penetration and faster blood clearance of unbound tracer [7]–[8]. The favorable in vivo kinetics of Affibody molecules permit high contrast imaging only a few hours after injections. More than 60 variants of Affibody molecules have been constructed and evaluated [9]. The most studied of these constructs are based on the Affibody molecule Z_HER2∶342_ binding the human epidermal growth factor receptor type 2 (HER2) with an affinity of 22 pM [10], or its derivatives [5]. HER2 receptors have been visualized using the anti-HER2 Affibody molecule in animal models with a contrast exceeding the contrast provided by any alternative imaging probe [11]. Clinical data have shown the feasibility of HER2 imaging using Affibody molecules in patients with metastasized breast cancer [12].

HER2 is a transmembrane tyrosine kinase receptor. HER2 dimerizes either with other members of HER family or homodimerizes with another HER2 receptor. Homo- or heterodimerization activates an intracellular tyrosine kinase domain leading to an intracellular signaling cascade increasing mitotic activity and suppressing apoptosis [13]. Malignant cells often overexpress the HER2 receptor, which provides an advantage in survival. The gene amplification and overexpression of HER2 have been linked to various cancer types, e.g. breast cancer, prostate cancer and ovarian cancer [14], [15]. Overexpression of the HER2 receptor is detected in 20–25% of the cases of primary breast cancer. The monoclonal antibody trastuzumab and the tyrosine kinase inhibitor lapatinib have been developed to target the HER2 receptors. Both trastuzumab [16] and lapatinib [17] improve survival of patients with metastatic HER2-expressing breast cancer. American Society of Clinical Oncology recommends evaluation of HER2 overexpression in each new or recurrent breast cancer to select patients who would benefit from trastuzumab or lapatinib therapy [18]. However, the existing biopsy-based methods of HER2 determination are invasive, and cannot address discordance of HER2 expression in primary tumors and metastases, as well as expression change during therapy [1], [2]. The use of molecular imaging, a non-invasive method for monitoring HER2 expression could replace biopsy sampling, providing a non-invasive, repeatable alternative allowing for simultaneous detection of HER2 in all metastases.

Labeling of Affibody molecules with positron-emitting radionuclides and the use of PET for imaging could further increase diagnostic accuracy because of the higher sensitivity and quantification accuracy provided by PET. One promising positron-emitting radionuclide is the generator-produced ^68^Ga (T_1/2_ = 68 min, E_β_+max = 1899 keV, 89% β+ yield). The rapid kinetics of Affibody molecules is compatible with the short half-life of ^68^Ga. Other potential advantages of ^68^Ga are reduced cost and easy availability since it is generator-produced.

**Figure 1:**
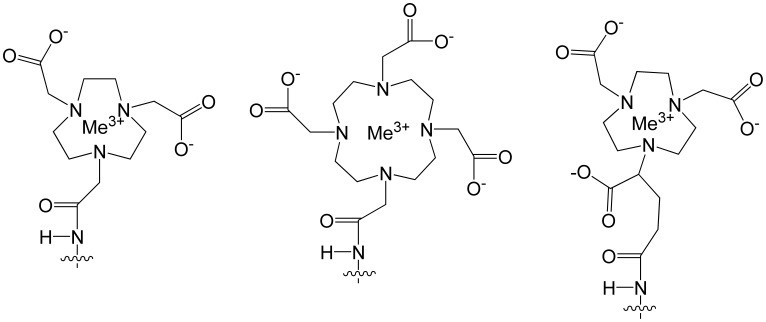
Metal complexes of the chelators 1,4,7-triazacyclononane-1,4,7-triacetic acid (NOTA), 1,4,7,10-tetraazacylododecane-1,4,7,10-tetraacetic acid (DOTA), and 1-(1,3-carboxypropyl)-1,4,7-triazacyclononane-4.7-diacetic acid (NODAGA) conjugated to N-terminal amino group via amide bonds.

Labeling chemistry has been previously shown to have profound influence on the tumor targeting and biodistribution profile of Affibody molecule. Small modification in peptide-based chelators dramatically altered blood clearance, liver uptake, route of excretion and renal retention of ^99m^Tc-labeled Affibody molecules [19]–[21]. The same effect was observed comparing different macrocyclic chelators for labeling of Affibody molecules with ^111^In [22], [23], [11]. Different radionuclides have also been shown to influence the imaging properties. Synthetic Affibody molecules labeled with both ^68^Ga and ^111^In using DOTA conjugated to the N-terminus had different clearance rates and different tumor-to-organ ratios [24].The homologous macrocyclic chelators, 1,4,7,10-tetraazacylododecane-1,4,7,10-tetraacetic acid (DOTA), 1,4,7-triazacyclononane-N,N,N-triacetic acid (NOTA) and 1-(1,3-carboxypropyl) -1,4,7- triazacyclononane-4,7-diacetic acid (NODAGA) (**Fig. 1**) have been shown to form stable complexes with ^68^Ga suitable for PET imaging [24]–[26]. Earlier, we have synthesized Affibody molecules with DOTA, NOTA and NODAGA conjugated at N-terminus [22]. We labeled these conjugates with ^111^In and evaluated their tumor targeting properties in xenograft-bearing mice [22]. The differences between the denticity, complex geometry and net charge of these chelator complexes with indium have been shown to have a significant influence on the biodistribution profile of ^111^In-labeled synthetic Affibody molecules and tumor-to-organ ratios [22]. These data suggest that it is possible to improve tumor-to-organ ratios, and in this way the imaging contrast, by selecting an optimal chelator for a particular nuclide.

The main hypothesis of the study was that different macrocyclic chelators have a different influence on the biodistribution of ^68^Ga-labeled synthetic Affibody molecules, and comparison of labeled synthetic HER2-targeting Affibody molecules using DOTA, NOTA and NODAGA conjugated to the N-terminus of the protein by amide bonds would enable us to select a ^68^Ga-labeled conjugate with the highest tumor-to-organ ratio (i.e. imaging contrast).

In addition, we intended to compare the biodistribution profiles of synthetic Affibody molecules labeled with ^68^Ga and ^111^In using DOTA, NOTA and NODAGA at the N-terminus to obtain structure-property knowledge for the design of future Affibody molecules for imaging of different molecular targets.

## Materials and Methods

### Materials

Buffers, including 0.1 M phosphate buffered saline (PBS), pH 7.5, 0.2 M ammonium acetate, pH 5.5, and 0.2 M citric acid were prepared using common methods from chemicals supplied by Merck (Darmstadt, Germany). High-quality Milli-Q^©^ water (resistance higher than 18 MΩ cm) was used for preparing the solutions. Buffers, which were used for labeling, were purified from metal contamination using Chelex 100 resin (Bio-Rad Laboratories, Richmond, USA). [^111^In]-indium chloride was purchased from Covidien (Hazelwood, US) as a solution in 0.05 M hydrochloric acid. The yield and radiochemical purity of the labeled affibody constructs were analyzed using 150–771 DARK GREEN, Tec-Control Chromatography strips from Biodex Medical Systems (New York, US). Distribution of radioactivity along the thin layer chromatography strips and SDS-PAGE gels was measured on a Cyclone Storage Phosphor System and analyzed using the OptiQuant image analysis software (Perkin Elmer, Wellesley, MA, USA). The accuracy of radio-ITLC analysis was cross-validated by SDS-PAGE. Ketalar [ketamine] (50 mg/mL, Pfizer, NY, USA), Rompun [xylazin] (20 mg/mL, Bayer, Leverkusen, Germany), and Heparin (5000 IE/mL, Leo Pharma, Copenhagen, Denmark) were obtained commercially.

Data on cellular uptake and biodistribution were analyzed by unpaired, two-tailed *t*-test using GraphPad Prism (version 4.00 for Windows GraphPad Software, San Diego, US) in order to determine any significant differences (P<0.05).

### Synthetic Affibody molecules: preparation and characterization

A synthetic variant of the Z_HER2∶342_ Affibody molecule_,_ here denoted Z_HER2:S1_, with the sequence AEAKYAKEMRNAYWEIALLPNLNNQQKRAFIRSLYDDPSQSANLLAEAKKLNDAQAPK-amide was synthesized by Fmoc/tBu-chemistry, manually conjugated with the DOTA, NOTA and NODAGA chelators, and purified by RP-HPLC as described earlier [22]. Biophysical characterization of the conjugates was performed by RP-HPLC, ESI-Q-TOF mass spectrometry, and circular dichroism, and the binding affinity to HER2 was determined by real-time biosensor (Biacore) analysis as described [22].

### Labeling chemistry and in vitro studies

For labeling with ^68^Ga, 30–50 µg of chelator-conjugated Z_HER2:S1_ was reconstituted in 60 µL 1.25 M sodium acetate buffer, pH 3.6. Fractions containing 500 µL were eluted from ^68^Ge/^68^Ga generator (50 mCi Eckert and Ziegler) with 0.1 M hydrochloric acid as eluent (prepared from 30% ultrapure HCl from Merck). The third fraction contained the maximum radioactivity and this was used for labeling. Approximately 2 MBq/µg Affibody molecule was added to the solution. The mixture was incubated at 95°C for 15 min, 5 µL of the mixture was mixed in 5 µL 0.2 M citrate buffer for 2 min. A small aliquot (1uL) was taken for analysis by radio-ITLC (DARK GREEN, Tec-Control Chromatography stripes, Biodiex Mecixal System), eluted with 0.2 M citric acid. After labeling the conjugates were purified using NAP-5 size-exclusion column, pre-equilibrated with PBS, 1.6 µL of the mixture was analyzed using ITLC.

To evaluate the stability of labeling, the ^68^Ga-labelled conjugates were incubated with 500-fold excess of EDTA for 1 h and then analyzed using ITLC. The experiments were performed in duplicates, for control experiment the conjugates were incubated with PBS.

Labeling of ^111^In was performed as previously described [22].

Binding specificity and cellular processing of ^68^Ga-DOTA-Z_HER2S:1_, ^68^Ga-NODAGA-Z_HER2:S1_ and ^68^Ga-NOTA-Z_HER2:S1_ was studied using HER2-expressing ovarian carcinoma SKOV-3 cells (1.6×10^6^ receptors/cells, [27]. The methods have been validated earlier [28]. ^68^Ga-DOTA-Z_HER2:S1_, ^68^Ga-NODAGA-Z_HER2:S1_ or ^68^Ga-NOTA-Z_HER2:S1_ with a protein concentrations of 27 pM was added to 6 dishes (10^6^ cells/dish). A 1000-fold excess of non-labeled recombinant Affibody molecule was added to 3 of these petri dishes 5 min before the labeled conjugate to saturate the receptors. The dishes were incubated for 1 h in a humidified incubator at 37°C. The media was collected, the cells were detached using trypsin-EDTA solution, and radioactivity was measured both in the media and cell suspension and percent of cell-bound radioactivity was calculated for both the pre-saturated and unsaturated cells.

Processing of ^68^Ga-DOTA-Z_HER2:S1_, ^68^Ga-NODAGA-Z_HER2:S1_ and ^68^Ga-NOTA-Z_HER2:S1_ by SKOV-3 cells during continuous incubation was studied according to a method described and validated by [28]. The labeled compounds (protein concentration of 27 pM) were added to petri dishes containing 10^6^ cells/dish. The cells were incubated at 37°C, in a humidified atmosphere containing 5% CO_2_. At predetermined time points (0.5, 1, 2 and 3 h after incubation started), the media from 3 dishes was collected and the cells were washed in ice-cold serum-free medium. The cells were then treated with 0.5 mL 0.2 M glycine buffer containing 4 M urea, pH 2.5, for 5 min on ice. The solution was collected, and the cells were washed additionally with 0.5 mL glycine buffer. The fractions were pooled together. The radioactivity of the acid wash fractions was considered to be membrane-bound radioactivity. The cells were then incubated at 37°C for at least 30 min with 0.5 mL 1 M NaOH. The alkaline solution was collected and the cell dishes were washed with an additional 0.5 mL NaOH and the alkaline fractions were pooled. The radioactivity in the alkaline fractions was considered as internalized. The radioactivity was measured with the automatic 1480 WISARD 3″ Gamma counter (Wallac). A percent of internalized radioactivity was calculated for each fraction at each time point.

### In vivo studies

Animal experiments have been performed according to national legislation on laboratory animal protection and were approved by the Ethic Committee for Animal Research of the Uppsala University (Permit Number: 48/11). Euthanasia was performed under Ropmpun/Ketalar anesthesia, and all efforts were made to minimize suffering. ^68^Ga and ^111^In labeled conjugates were compared using a dual label study was performed to improve the statistical power and to minimize the number of tumor-bearing animals, as it was described by Honarvar and co-workers [29].

Targeting properties of different conjugates were compared in female BALB/c nu/nu mice (15 weeks old, mean weight of 18±1 g) carrying SKOV-3 xenograft. The cells (10^7^ cells per mouse) were subcutaneously implanted in the right hind leg 5 weeks before experiment. At the time of experiment, an average tumor weight was 0.5±0.3 g. A mixture of 10 kBq ^111^In-DOTA-Z_HER2:S1_ and 380 kBq ^68^Ga-DOTA-Z_HER2:S1_ in 100 µL PBS each were injected in two groups of mice (four mice each). The total injected amount protein dose was adjusted to 3 µg/animal by adding unlabeled protein. In order to saturate HER2 receptors in tumors, one group of mice was injected with 1000 µg of unlabeled recombinant Z_HER2∶342_ Affibody molecules. The same was done for the other conjugates.

The mice were euthanized at 2 h p.i. by an intra-peritoneal injection of Ketalar-Rompun solution (20 µl of solution/g body weight: Ketalar, 10 mg/ml; Rompun, 1 mg/ml) followed by heart puncture with a syringe rinsed with heparin (5,000 IE/ml) and exsanguination. Blood and organ samples: lung, liver, spleen, kidneys, tumor, muscle, bone, gastrointestinal tract (with content) and the remaining carcass were collected and weighed. The whole spectra for each sample as well as spectra of injected solution standards were recorded immediately after injection and 17 h later (after complete decay of ^68^Ga). The data were corrected for background radiation, gamma spectrometer dead time percentage during each measurement, and decay during measurement. Based on the second measurement, organ uptake values for ^111^In were calculated as percent of injected activity per gram of tissue (%IA/g), except for the gastrointestinal tract and the remaining carcass, which were calculated as %IA per whole sample. Thereafter, indium counts were corrected for decay (using an ^111^In sample as standard) and subtracted from counts obtained during first measurement (immediately after dissection). The resulted values presented radioactivity of ^68^Ga in each organ during the first measurement. These values were used to calculate biodistribution of ^68^Ga-labeled tracer.

### Imaging

The conjugate with best tumor to organ ratio in this case ^68^Ga-NODAGA-Z_HER2:S1_was imaged to confirm the capacity for HER2 visualization. One SKOV-3 bearing mouse was injected with 2.5 MBq of ^68^Ga-NODAGA-Z_HER2:S1_ (3.3 µg peptide). Immediately before imaging (2 h pi), the animal was sacrificed and the urine bladder was dissected to avoid interfering activity in urine close to tumor xenograft and kidneys. The PET/CT imaging was performed in The Triumph™ Trimodality system (Gamma Medica, Inc) a fully integrated SPECT/PET/CT hardware and software platform optimized for small animals. The acquisition time was 60 min. The PET data was reconstructed into a static image using a MLEM 2D algorithm (10 iterations). The CT raw file was reconstructed using Filter Back Projection (FBP). PET and CT dicom files were analyzed using PMOD v 3.12 software (PMOD Technologies Ltd, Zurich, Switzerland).

## Results

### Peptide synthesis and biophysical characterization

Data concerning the chelator-conjugated Affibody molecules are summarized in Table 1. All variants had a purity of over 96%. The melting points were in the range of 63–64°C and the affinities (apparent dissociation constant at equilibrium) were in the range of 90–140 pM [22].

**Table 1:** Biophysical characteristics of the Affibody conjugates [22].

Conjugate	Purity	T_m_	K_D_
DOTA-Z_HER2:S1_	98%	64°C	133 pM
NOTA-Z_HER2:S1_	97%	63°C	140 pM
NODAGA-Z_HER2:S1_	99%	63°C	90 pM

### Labeling chemistry and in vitro studies

The labeling with ^68^Ga in the described conditions provided a high yield for all three conjugates. Average radiochemical yields for the DOTA and NODAGA conjugates were approximately 95%, and the labeling efficiency of the NOTA-conjugate was somewhat lower (87.4±10.4% ) (**Table 2**). After purification using a disposable autoclavable NAP-5 size-exclusion column a typical radiochemical purity of over 97% was provided for all three conjugates (**Table 2**). A challenge with 500-fold excess EDTA during 1 h demonstrated no measurable release of radioactivity from conjugates (Table 2), which demonstrated a high stability of all labels.

**Table 2:** Labeling yield (decay corrected), radiochemical purity and stability of ^68^Ga-labeled Affibody molecules.

Conjugate	Yield	Purity	Stability under EDTA challenge* (EDTA)	Stability under EDTA challenge* (control)
^68^Ga-DOTA-Z_HER2:S1_	94.6±1.0%	99±2.1%	99.5±0.1%	99.2±0.1%
^68^Ga-NOTA-Z_HER2:S1_	87.4±10.4%	98.3±2.1%	99.7±0.0%	99.5±0.1%
^68^Ga-NODAGA-Z_HER2:S1_	94.6±4.7%	99.3±0.2%	99.5±0.1%	99.4±0.1%

* To evaluate stability, conjugates were incubated with 500-fold excess of EDTA for 1 h and then analyzed using ITLC. Control samples were incubated in PBS. The experiments were performed in duplicates. The data present an average conjugate-associated radioactivity and maximum error.

**Figure 2:**
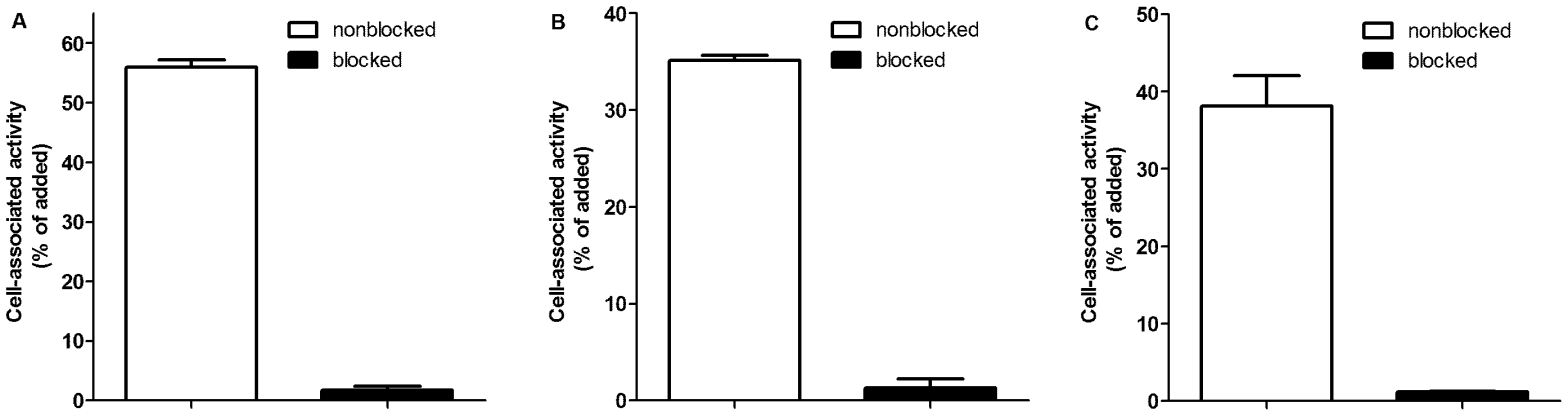
In vitro specificity test of ^68^Ga-NODAGA-Z_HER2:S1_ (A), ^68^Ga-NOTA-Z_HER2:S1_ (B) and ^68^Ga-DOTA-Z_HER2:S1_ (C) binding to HER2 expressing SKOV-3 cells. **Dishes containing cells were incubated with 27 pM radiolabeled conjugated.** One group of dishes was pre-saturated with 1000-fold excesses of non-labeled Z_HER2:S1_ to block the binding site of HER2. Cell-associated radioactivity was calculated as percentage of total added radioactivity. Data are presented as mean values for three cell dishes and standard deviations.

The results of the binding specificity tests are presented in Figure 2. There was significantly lower binding (p<0.001) of all three conjugates to the HER2-expressing SKOV-3 cells (**Fig. 2**) treated with a large excess of the recombinant Z_HER2∶342_ Affibody molecule. This demonstrates saturability of binding and suggests receptor-mediated binding of ^68^Ga-DOTA-Z_HER2:S1_, ^68^Ga-NODAGA- Z_HER2:S1_ and ^68^Ga-NOTA-Z_HER2:S1_ to HER2-expressing cells.

**Figure 3:**
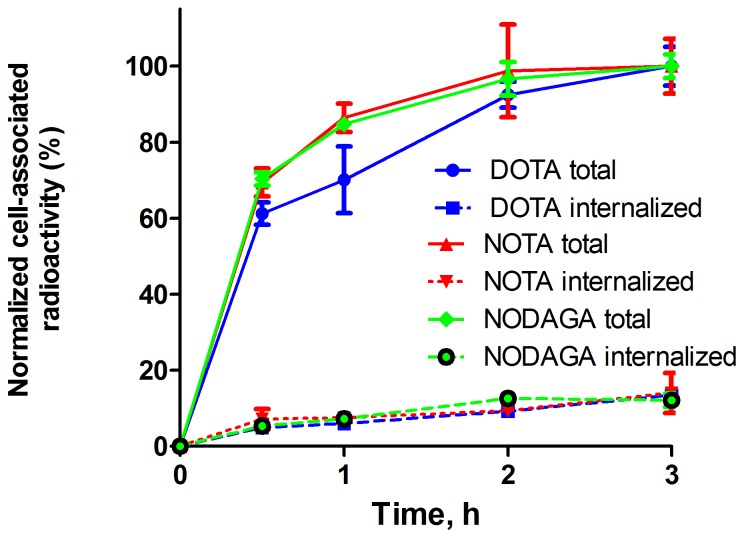
Cellular processing of ^68^Ga-NODAGA-Z_HER2:S1_, ^68^Ga-NOTA-Z_HER2:S1_ and ^68^Ga-DOTA-Z_HER2:S1_ by HER2-expressing cells SKOV-3 in vitro. Cells were incubated with labeled compound at 37°C. Cell bound activity is normalized to the maximum uptake. Data are presented as mean values for three cell dishes and standard deviations. Error bars might be smaller than the symbols.

Data for the cellular processing of HER2-expressing SKOV-3 cells are presented in **Figure 3**. The processing patterns were typical of Z_HER2∶342_ and its derivatives and were very similar for all three conjugates (difference within accuracy of the method). The internalization was slow for all conjugates, although the internalized fraction of activity was increasing with time. After 3 h-long incubation, the internalized fraction was 13±5% for^ 68^Ga-DOTA-Z_HER2:S1_, 14±2% for ^68^Ga-NODAGA-Z_HER2:S1_ and 12±5% for ^68^Ga-NOTA-Z_HER2:S1_. The slow internalization of ^68^Ga-labeled Affibody molecules was in agreement with the data obtained earlier for their ^111^In-labeled counterparts [22].

### *In vivo* studies

**Figure 4:**
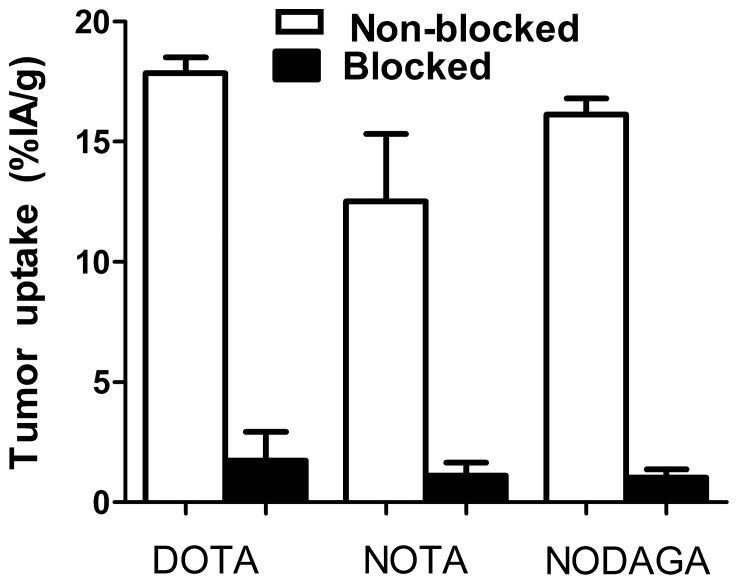
Specificity of targeting of SKOV-3 xenografts in BALB/C nu/nu mice using ^68^Ga-DOTA-Z_HER2:S1_, ^68^Ga-NOTA-Z_HER2:S1_ and ^68^Ga-NODAGA-Z_HER2:S1_ at 1h after injection. The total injected radiolabeled peptide dose per mouse was 3 µg (0.42 nmol). The blocked group was subcutaneously pre-injected with an excess amount (1000 µg, 150 nmol) of non-labelled Z_HER2∶342_ to saturate binding sites of HER2. Results are expressed as percentage of injected activity per gram of tissue (%IA/g) and presented as mean values for four mice and standard deviations. Uptake of radioactivity was significant lower (p<0.0005) for pre-saturated SKOV-3 tumors.

Data concerning comparative in vivo study are presented in Figure 4 and Tables 3 and 4.

**Table 3:** Comparative biodistribution of NODAGA-Z_HER2:S1_, NOTA-Z_HER2:S1_ and DOTA-Z_HER2:S1_ labelled with gallium-68 and indium-111 after intravenous injection in female BALB/C nu/nu mice bearing SKOV-3 xenografts.

	DOTA-Z_HER2:S1_		NOTA-Z_HER2:S1_		NODAGA-Z_HER2:S1_	
	^68^Ga	^111^In	^68^Ga	^111^In	^68^Ga	^111^In
Blood	0.7±0.1^d^	0.47±0.05^a^	0.3±0.0	0.64±0.08^b^	0.28±0.05^f^	0.5±0.1^c^
Lung	0.7±0.2^d^	1.0±0.3^a^	0.5±0.1	0.9±0.2	0.6±0.2	1.9±1.7
Liver	3.3±0.4	2.8±0.3^a^	3.7±0.3^e^	6.6±0.6^b^	2.4±0.6	3±1^c^
Spleen	1.5±0.3^d^	1.3±0.4	0.7±0.2	1.1±0.2	0.5±0.1^f^	1.1±0.3^c^
Kidney	295±31	305±32^a^	312±11	203±8^b^	294±22	291±32
Tumor	17.9±0.7^d^	20.9±0.7^a^	12.5±2.8	9±1^b^	16.1±0.7^f^	14.4±0.9^c^
Muscle	0.07±0.03	0.23±0.09^a^	0.09±0.03	0.06±0.03	0.15±0.03^f^	0.1±0.2^c^
Bone	0.09±0.05	0.8±0.1^a^	0.3±0.2	0.22±0.07	0.49±0.01^f^	0.2±0.6
GI tract*	0.7±0.1^d^	0.5±0.3	0.46±0.06	0.58±0.05^b^	0.7±0.2	0.9±0.2^c^
Carcass*	4.8±1.4	4±3	4.7±1.5	5±1	5.1±2.6	7±2^c^

Data are presented as an average % IA/g and standard deviation for four mice. Data concerning bone from one mice injected with ^68^Ga-NOTA-Z_HER2:S1_ and bone and muscle form one mice injected with ^68^Ga-NODAGA-Z_HER2:S1_were excluded due to low counts.

a significant difference (p<0.05) between ^68^Ga-DOTA-Z_HER2:S1_ and ^111^In-DOTA-Z_HER2:S1_.

b significant difference (p<0.05) between ^68^Ga-NOTA-Z_HER2:S1_ and ^111^In-NOTA-Z_HER2:S1_.

c significant difference (p<0.05) between ^68^Ga-NODAGA-Z_HER2:S1_ and ^111^In-NODAGA-Z_HER2:S1_.

d significant difference (p<0.05) between ^68^Ga-DOTA-Z_HER2:S1_ and ^68^Ga-NOTA-Z_HER2:S1_.

e significant difference (p<0.05) between ^68^Ga-NOTA-Z_HER2:S1_ and ^68^Ga-NODAGA-Z_HER2:S1_.

f significant difference (p<0.05) between ^68^Ga-NODAGA-Z_HER2:S1_ and ^68^Ga-DOTA-Z_HER2:S1_.

* Data for gastrointestinal (GI) tract and carcass are presented as %IA per whole organ.

**Table 4:** Tumor-to-organ ration data 2h after injection for NODAGA-Z_HER2:S1_, NOTA-Z_HER2:S1_ and DOTA-Z_HER2:S1_ in mice bearing SKOV-3 xenografts.

	DOTA-Z_HER2:S1_		NOTA-Z_HER2:S1_		NODAGA-Z_HER2:S1_	
	^68^Ga	^111^In	^68^Ga	^111^In	^68^Ga	^111^In
Blood	28±4	44±3^a^	42±11	13±4^b^	60±10^f^	31±4^c^
Lung	25±4	22±5^a^	26±9	8±5^b^	31±12	15±1^c^
Liver	5.5±0.6^d^	7.4±0.7^a^	3.4±0.6^e^	1.2±0.2^b^	7±2	5±2^c^
Spleen	12±2	16.4±3.7^a^	19±6^e^	7±2^b^	36±10^f^	15±4^c^
Kidney	0.1±0.0^d^	0.07±0.01^a^	0.04±0.10^e^	0.04±0.01^b^	0.06±0.01	0.05±0.00^c^
Muscle	297±109^d^	98±32	80±15	58±22^b^	100±25^f^	161±58
Bone	255±185	25±3^a^	25±4	13±2^b^	35±9	72±57

Data are presented as an average and standard deviation for four mice. Data concerning bone from one mice injected with ^68^Ga-NOTA-ZHER2:S1 and bone and muscle form one mice injected with ^68^Ga-NODAGA-ZHER2:S1were excluded due to low counts.

a significant difference (p<0.05) between ^68^Ga-DOTA-Z_HER2:S1_ and ^111^In-DOTA-Z_HER2:S1_.

b significant difference (p<0.05) between ^68^Ga-NOTA-Z_HER2:S1_ and ^111^In-NOTA-Z_HER2:S1_.

c significant difference (p<0.05) between ^68^Ga-NODAGA-Z_HER2:S1_ and ^111^In-NODAGA-Z_HER2:S1_.

d significant difference (p<0.05) between ^68^Ga-DOTA-Z_HER2:S1_ and ^68^Ga-NOTA-Z_HER2:S1_.

e significant difference (p<0.05) between ^68^Ga-NOTA-Z_HER2:S1_ and ^68^Ga-NODAGA-Z_HER2:S1_.

f significant difference (p<0.05) between ^68^Ga-NODAGA-Z_HER2:S1_ and ^68^Ga-DOTA-Z_HER2:S1_.

The results of in vivo specificity tests are presented in the Figure 4. The tumor uptake of all ^68^Ga-labeled conjugates was significantly (p<0.0005) lower after saturation of HER2 in xenografts by pre-injection of a large molar excess of non-labeled Z_HER2∶342_. The saturable uptake of the conjugates in tumors suggests that the tumor accumulation was HER2-specific. The pre-injection of Z_HER2∶342_ caused no significant difference in uptake in any other organs (data not shown). Uptake of ^111^In-labelled conjugates was also HER2-specific (data not shown).

All conjugates demonstrated rapid clearance from blood and low uptake in normal organs (except from kidneys) and tissues (Table 3). Uptake of radioactivity in the gastrointestinal tract (with content) was low, indicating that hepatobiliary excretion played a minor role in the clearance. The high uptake in kidney suggests that the conjugates were cleared by glomerular filtration with subsequent re-absorption in kidneys. The chelators had no influence on the renal retention of radioactivity of the ^68^Ga-labeled conjugates, but there was significant difference in the renal retention of the ^68^Ga- and ^111^In-labeled counterparts for NODAGA-Z_HER2:S1_ and DOTA-Z_HER2:S1._

Among the ^68^Ga- labeled conjugates, the tumor uptake was the highest for ^68^Ga-DOTA-Z_HER2:S1_ followed by ^68^Ga-NODAGA-Z_HER2:S1._ The tumor uptake of ^68^Ga-NOTA-Z_HER2:S1_ was the lowest. The influence of chelator on tumor uptake was similar for the ^111^In-labeled tracers (^111^In-DOTA-Z_HER2:S1_>^111^In- NODAGA-Z_HER2:S1_>^111^In- NOTA-Z_HER2:S1_), and was appreciably more clearly pronounced, i.e. the difference between the conjugates was bigger. There was no clear pattern regarding the influence of nuclide on tumor uptake. The tumor uptake was significantly higher for ^68^Ga-NODAGA-Z_HER2:S1_ than for ^111^In-NODAGA-Z_HER2:S1_, and for ^68^Ga-NOTA-Z_HER2:S1_ than for ^111^In-NOTA-Z_HER2:S1_, while ^68^Ga-DOTA-Z_HER2:S1_ had lower tumor uptake than ^111^In-DOTA-Z_HER2:S1_.

^68^Ga-NODAGA- Z_HER2:S1_ had the lowest retention in blood (0.28±0.5%IA/g) and lower uptake in organs such as liver (2.4±0.6%IA/g), spleen (0.5±0.1%IA/g), kidney (294±22%IA/g), muscle (0.15±0.03%IA/g) and bone (0.49±0.01%IA/g) than the ^68^Ga-labeled NOTA and DOTA conjugates. Comparing ^68^Ga-NODAGA-Z_HER2:S1_ to ^111^In-NODAGA-Z_HER2:S1_, there was significantly lower retention in blood and lower uptake in liver, GI tract and carcass for the ^68^Ga-labeled variant. Comparison of the other conjugates demonstrated a strong influence of the radionuclide on uptake in normal organs and tissues when the same chelator was used. Notably, the liver uptake for ^111^In-labeled NOTA-Z_HER2:S1_ was significantly higher than for the ^68^Ga-labeled conjugate (6.58±0.56 vs. 3.73±0.32), which has not been shown earlier. Still, ^68^Ga-NOTA- Z_HER2:S1_ had the highest liver uptake among the ^68^Ga-labeled tracers.

The tumor-to-organ ratios are presented in Table 4. ^68^Ga-NODAGA-Z_HER2:S1_ had the highest tumor-to-blood, tumor-to-liver, tumor-to-spleen and tumor-to-kidney ratio among the ^68^Ga-labeled variants. There were significantly (p<0.05) higher values for ^68^Ga-NODAGA-Z_HER2:S1_ than for ^68^Ga-DOTA-Z_HER2:S1_ for tumor-to-blood (60±10 vs. 28±4), tumor-to-spleen (34±10 vs. 12±2) and tumor-to-muscle (100±25 vs. 296±108) ratios. The tumor-to-organ ratios were significantly higher in the case of ^68^Ga-NODAGA- Z_HER2:S1_ than in the case of ^68^Ga-NOTA- Z_HER2:S1_ for liver (7±2 vs. 3±1), spleen (35±10 vs. 19±6) and kidney (0.1±0.01 vs. 0.04±0.01).

Images acquired after injecting ^68^Ga-NODAGA-Z_HER2:S1_ in mice bearing SKOV-3 xenografts confirmed the ability to visualize the HER2-expressing tumors using PET. In agreement with the results of the biodistribution study, high accumulation of radioactivity was observed in the kidney. There was no noticeable uptake of radioactivity in other organs.

## Discussion

Identification of patients with tumors expressing particular molecular therapeutic targets may help in the selection of an appropriate targeting therapy and make their treatment more personalized. The use of *in vivo* molecular imaging might provide a non-invasive, repetitive method for determination of target expression levels in disseminated cancer during the course of disease [30]. Application of PET might further increase the accuracy of molecular diagnostics due to the possibility of visualization of small metastases. Although ^18^FDG-PET is frequently used for oncology imaging, it provides only information about cell glucose metabolism, and can be utilized for therapy monitoring, but it is not informative in detection of other molecular abnormalities. Development of new PET tracers, which can give quantitative information about expression of molecular therapeutic targets by malignant cells, is therefore desirable. PET imaging using a HER2-targeting Affibody molecule labeled with ^68^Ga has shown sufficient quantification accuracy for the detection of different levels of HER2 expression in murine xenografts [31]. However, further increase of sensitivity is desirable to enable imaging of small metastases. It has to be noted that besides HER2-imaging agents, there are currently several new Affibody molecules targeting other cancer-associated receptors, e.g. EGFR [32], IGF-1R [33], PDGF-Rβ [34] and HER3 [35], under development. Information about optimal labeling strategies would be useful for further improvement of these imaging probes.

The HER2-targeting Affibody molecule is the most studied Affibody tracer. It has been labeled with several radionuclides using different labeling methods [5]. Experiments with HER2-targeting Affibody molecules have provided substantial information on how different labeling strategies can influence the targeting properties. Furthermore, the HER2-targeting Affibody molecules offer a suitable model to study general features of the biodistribution of Affibody molecules, as there is no noticeable HER2-expression in normal tissues [36]. Hence non-target specific interactions with normal tissues, which influence the biodistribution of this kind of tracers, can be studied using HER2-binding Affibody molecules, without interference with target-specific interactions. Factors such as radionuclide, chelator and Affibody scaffold modifications can be evaluated using HER2-binding Affibody molecules.

Previous studies have demonstrated that small changes in the chelator structure and conjugation linker, as well as the site of its conjugation to the Affibody molecule, affected clearance rate from blood, and influenced uptake in normal tissues and in tumors [8], [11], [23]. Modification of the chelator properties and its positioning can increase the tumor-to-organ ratios appreciably, around two-fold [8], [37]. Additionally, the influence of chelator on the biodistribution profile also depends on the attached radionuclide [24], [38]. This suggests that labeling chemistry (i.e. the combination of the chelator, the linker for its attachment to an imaging probe, the conjugation site and the radionuclide) can be used not only for coupling of a nuclide to a targeting protein, but also as a mean to modify its biodistribution. This opens up ways to further improve the imaging properties through alteration of the chemical and physicochemical features of the targeting molecule. These findings could also be valuable for development other scaffold proteins with similar size that are evaluated for molecular imaging, e.g. DARPins [39], knottins [40], and fibronectin domains [41].

In the current study, three different chelators (DOTA, NOTA and NODAGA) were site-specifically coupled via amide bond to the N-terminus of the HER2-targeting Affibody molecule Z_HER2:S1_ and labeled with ^68^Ga. The Affibody molecule used in this study was synthetically produced and the chelators were coupled as the last step of the peptide synthesis. The conjugates were evaluated in vitro and compared with each other and to their ^111^In-labeled counterparts in vivo using a dual label protocol.

The results demonstrated high-yield, site-specific, stable labeling of Affibody molecules with ^68^Ga by the macrocyclic chelators DOTA, NOTA and NODAGA, conjugated to the N-terminus. The labeled conjugates retained binding specificity to the HER2-expressing SKOV-3 cells (Figure 2). The cellular processing study of ^68^Ga-DOTA-Z_HER2S:1_, ^68^Ga-NODAGA-Z_HER2:S1_ and ^68^Ga-NOTA-Z_HER2:S1_ showed slow internalization and similar processing patterns for all three conjugates, suggesting that there is no substantial influence of the chelators on the cellular processing of the conjugates.

All the conjugates targeted specifically HER2-expressing xenografts in mice, as the tumor uptake was reduced by pre-saturation of HER2 via pre-injection of large excess of non-labeled Z_HER2∶342_ (Figure 4). There was a clear influence of the labeling chemistry on the biodistribution (Table 3). The EDTA challenge has demonstrated stable chelation of ^68^Ga by all chelators (Table 2). A number of previous studies (see e.g. [21], [24]) has shown that the protein backbone is stable in blood. Most likely, the differences in biodistribution are caused neither by loss of nuclides from chelators nor by different pattern of blood proteases cleavage products. Therefore, we believe that the biodistribution differences are due to different off-target interactions of the conjugates. Interestingly, for ^111^In-labeled Affibody molecules the influence was more pronounced than for ^68^Ga-labeled ones (Table 3). There were significant nuclide- and chelator-dependent differences in tumor uptake, although the affinities (dissociation constants at equilibrium) were rather close for all conjugates, in the range of 90 to 140 pM (Table 1). Earlier, we have investigated effect of affinity of recombinant anti-HER2 Affibody molecules on uptake in tumors with high and low expression of HER2 [27]. All conjugates in that study were site-specifically labeled with ^111^In in the same way, at C-terminal cysteine using maleimido derivatives of DOTA. The SKOV-3 xenografts (as in the current study) were used as a tumor model with high target expression. We found that uptake in SKOV-3 xenografts at 4 h after injection did not depend on affinity in the affinity range of 116–3804 pM [27]. Results of the current study indicate that off-target interactions of an imaging agent (determined by influence of a chelator and a label nuclide) might have stronger impact on its tumor uptake than its affinity. For example, the data for the ^111^In-labeled NOTA conjugate were in agreement with previous results [22] showing unfavorable high liver uptake. This “liver barrier” might be the reason for lower accumulation of NOTA-conjugated Affibody molecules in tumors. The high liver uptake (3.7±0.3% IA/g for ^68^Ga and 6.6±0.6% IA/g for ^111^In-labeled NOTA conjugates) can possibly be explained by the localized positive charge at the N-terminus formed by complexation of three-valent ^68^Ga and ^111^In with the NOTA chelator. An increase of hepatic uptake with increasing localized N-terminal positive charge has been observed earlier for ^99m^Tc-labeled anti-HER2 Affibody molecules [19], [42], [43]. However the magnitude of liver uptake was lower for the ^68^Ga-labeled NOTA conjugate than for the ^111^In-labeled. The difference in liver uptake between NOTA-, DOTA- and NODAGA conjugates was less for ^68^Ga- than for ^111^In-labeled conjugates.

**Figure 5:**
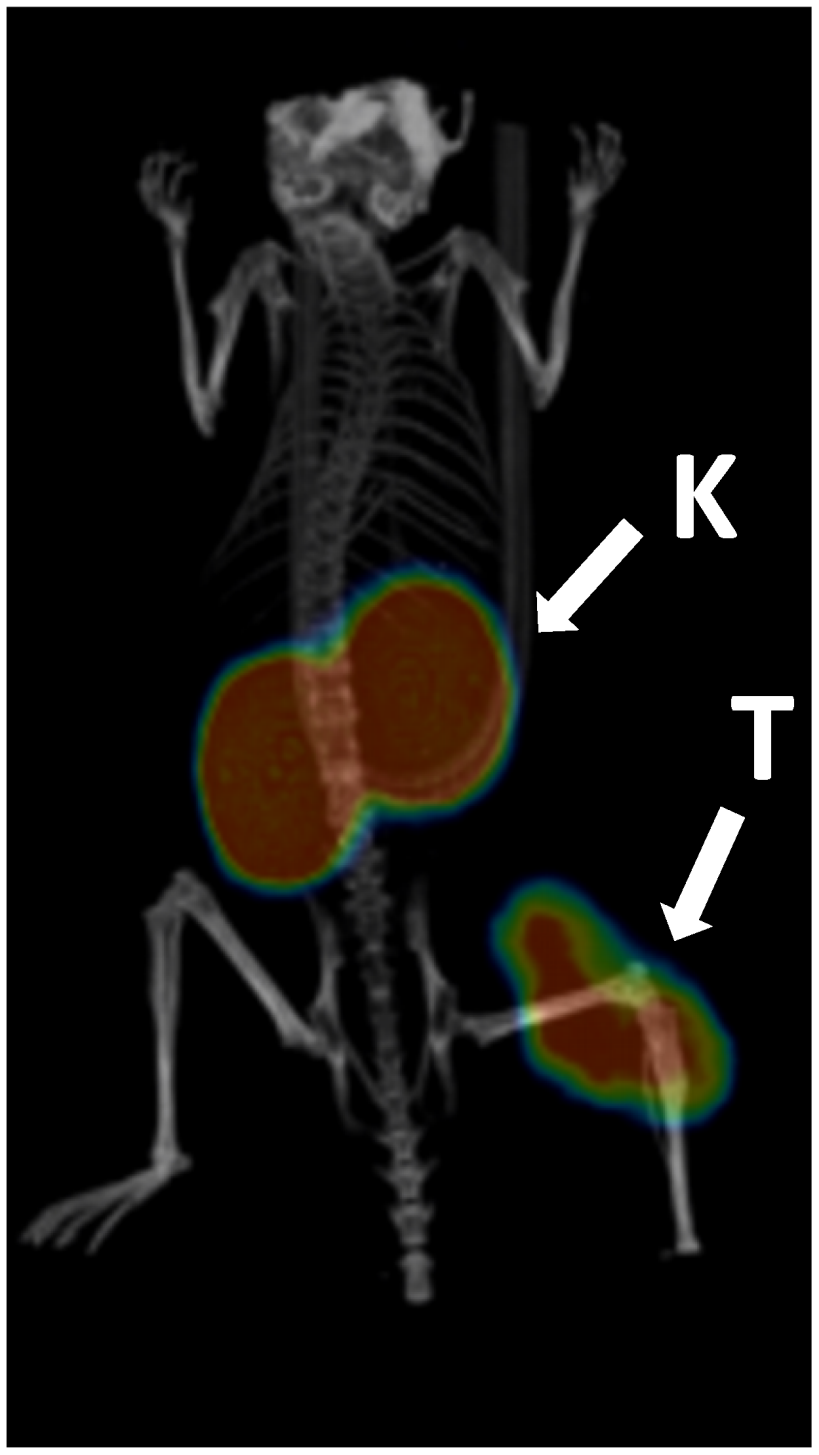
MicroPET/CT imaging of HER2 expression in SKOV3 xenografts in BALB/C nu/nu mice using ^68^Ga-NODAGA-Z_HER2:S1_. The image was acquired 2 h after administration of the tracer. Arrows point at tumor (T) and kidneys (K).

The results of this study suggest that NODAGA provides the best imaging properties among synthetic Affibody molecules conjugated with macrocyclic chelators via amide bond at the N-terminus and labeled with ^68^Ga. ^68^Ga-NODAGA-Z_HER2:S1_ provided more than 2-fold higher tumor-to-blood and tumor-to-spleen ratios than ^68^Ga-DOTA-Z_HER2S:1_. Compared to ^68^Ga-NOTA-Z_HER2:S1,_^68^Ga-NODAGA-Z_HER2:S1_ gave 2-fold higher tumor-to-liver and tumor-to-spleen ratios (Table 4). Since the liver is a major metastatic site for several cancers, a higher contrast in imaging of liver metastases is important. Apparently, ^68^Ga-NODAGA-Z_HER2:S1_ may improve detection of liver metastases using PET. As early as 2 hours pi ^68^Ga-NODAGA-Z_HER2:S1_ showed the capacity to image HER2-expressing tumor xenografts using micro-PET (Figure 5). Regarding the influence of radionuclide, NODAGA-Z_HER2:S1_ labeled with ^68^Ga had significantly lower uptake for the majority of the tissues (blood, liver, spleen, GI-tract and carcass) (table 3) and significantly higher uptake in tumor than the ^111^In-labeled variant.

Although In^3+^ and Ga^3+^ are both trivalent metals, they differ appreciably in ionic radii. Accordingly, there are appreciable differences in the structures of their complexes with DOTA [44] and NOTA [45]. Although there are no experimental structured data for complexes with NODAGA, it is reasonable to suppose that their structures will be similar to structures of NOTA complexes. The data for the homologous chelator NODASA (1,4,7-triazacyclononane-1-succinic acid-4,7-diacetic acid) support this assumption [46]. Different structures of chelates would result in different local charge distribution, as well as preferable conformations of the N-termini. It is conceivable that this would translate into different character of off-target interactions and affect the biodistribution. Several studies have demonstrated that substitution of ^111^In for ^68^Ga in short DOTA-conjugated peptides have altered both uptake in normal organs and tumors [47]–[50]. However, there was very little information about the effect of such or similar substitutions for larger scaffold proteins. Recently, we have studied radiofluorination of recombinant Affibody molecules using chelation of ^18^F-aluminium fluoride by maleimido derivative of NOTA conjugated to cysteine at C-terminus [51]. As comparators, ^111^In and ^68^Ga were used. In that study, ^68^Ga-labeled conjugate had also rapider blood clearance and provided higher tumor-to-blood ratio. The liver uptake was lower for ^68^Ga-labeled conjugate as well. However, a magnitude of the hepatic uptake was appreciably lower (2–3% IA/g) than in the current study. This suggest, that influence of a chelator on biodistribution depends on its position on an Affibody scaffold (N-or C-terminus), and might have a synergistic effect with the influence of surrounding amino acids. Besides, a linker for chelator conjugation to a scaffold protein might play a role due to its impact of overall and local lipophilicity. In another recent study, maleimido derivatives of DOTA and NODAGA chelators were used for site-specific labeling (C-terminus) of recombinantly produced Affibody molecules with ^111^In and ^68^Ga [52]. Again, a clear influence of radionuclide on biodistribution and targeting was observed. In agreement with the data from the current study, the difference was smaller for ^68^Ga than for ^111^In. NODAGA was found to be superior chelator for ^68^Ga even in the case of C-terminal placement. However, further investigations are required to evaluate if this is the true for other scaffold proteins. One particular issue is influence of chelators on bone uptake. Data form this and other [51] studies suggest that the bone uptake depends on combination of a radionuclide and a chelator. However, molecular mechanisms of such influence (except from chelate stability) are not quite clear. On the other hand, the bone uptake is essential because it determines sensitivity of bone metastases detection and might be a dose limiting factor for therapy in theranostics setting.

Creating a single tracer for both SPECT and PET imaging would be very desirable. In reality, this is complicated since nuclide exchange alters the imaging contrast, and the same construct might not be optimal for both ^111^In and ^68^Ga. The short half-life of ^68^Ga requires a tracer providing a maximum contrast within 2–3 hours after injection. This tracer might be not optimal for ^111^In at this time point. However, the longer half-life of ^111^In provides certain flexibility in selection of imaging time, and a later time point might be chosen when the tracer would provide better contrast due to clearance from normal tissues.

In conclusion, this paper has demonstrated that macrocyclic chelators conjugated to the N-terminus have a substantial influence on the biodistribution of synthetic Affibody molecules labeled with ^68^Ga. This can be used to enhance imaging contrast of PET imaging using Affibody molecules and improve sensitivity of molecular imaging. The study has demonstrated an appreciable difference in the influence of chelator for ^68^Ga and ^111^In. This information should be taken into account during development of scaffold protein-based probes for molecular imaging.
